# Managing Memory - Optimising Anti-Dementia Medication Prescribing in an Inpatient Setting

**DOI:** 10.1192/bjo.2026.11669

**Published:** 2026-06-30

**Authors:** Sharvin Chougule, Ting-Yu Chen, Agata Gwincinska, Louisa Marchant-Rutherford, Josef Mahdi

**Affiliations:** 1 University Hospitals Sussex NHS Foundation Trust, Worthing, United Kingdom; 2 Sussex Partnership NHS Foundation Trust, Worthing, United Kingdom

## Abstract

**Aims:**

Sub-optimal prescribing of anti-dementia medications may compromise the cognition of patients with dementia. The aim of this audit was to assess the prescribing of anti-dementia medications, including Donepezil, Rivastigmine and Memantine, for patients diagnosed with dementia against local and national guidelines in relation to three domains: appropriate indication for dementia subtype, optimal dosage titration and recommended physical health monitoring. The target was to achieve a minimum of 90% adherence to guidelines for each domain.

**Methods:**

This retrospective clinical audit was based at the Forget Me Not Unit, a specialist dementia intensive care unit in Worthing. The first audit cycle collected data from twenty patients discharged between February and May 2025. Data obtained from electronic medical records included dementia subtype and severity, pre-admission and inpatient-initiated anti-dementia medications, titration regimes, pulse checks and renal function profiles. Results were compared against Trust and Maudsley prescribing guidelines which served as standards. An action plan was subsequently implemented which included raising staff awareness of the audit, disseminating its results and placing visible audit reminders on ward lists. A second cycle was commenced in November 2025 to February 2026 which examined the data of twenty discharged patients following implementation of the action plan.

**Results:**

In the first cycle, thirteen out of twenty patients were prescribed pre-admission anti-dementia medications (65%) whilst seven out of twenty patients were candidates for an inpatient-initiated anti-dementia medication given their dementia subtype (35%). Six of these patients were offered an anti-dementia prescription of which five were clearly indicated (83%). All prescriptions were titrated according to guidelines but recommended physical health monitoring was only completed for five patients (83%). Similar results were demonstrated in the second cycle. Eleven patients were prescribed an anti-dementia medication prior to their admission (55%), six patients were candidates for an anti-dementiamedication (30%) but only four patients were prescribed an indicated drug during their admission (67%). However, all inpatient-initiated anti-dementia medications were titrated and monitored according to standards.

**Conclusion:**

Anti-dementia medications are licensed treatments for dementia but may be underutilised as evidenced by this audit. Although the target was achieved by the second cycle in two out of three domains, the indication of anti-dementia prescriptions was not fully adhered to against guidelines. Further audit cycles and tailored action plans could raise the standards of anti-dementia prescriptions and thereby improve patient safety and care.

